# Benign schwannoma of posterior mediastinum accompanied by bloody pleural effusion misdiagnosed as solitary fibrous tumor: A case report

**DOI:** 10.22088/cjim.10.4.468

**Published:** 2019

**Authors:** Ramin Nosrati, Diana Anissian, Farangis Ramezani, Majid Sharbatdaran

**Affiliations:** 1 Department of Surgery, Ayatollah Rouhani Hospital, Babol University of Medical Sciences, Babol, Iran; 2 Student Research Committee, Babol University of Medical Sciences, Babol, Iran; 3 Department of Pathology, Ayatollah Rouhani Hospital, Babol University of Medical Sciences, Babol, Iran

**Keywords:** Schwannoma, Pleural effusion, Solitary fibrous tumor, Thoracic surgery, Video-assisted

## Abstract

**Background:**

Schwannoma is a peripheral nerve sheath tumor originating from schwann cells. It is the most common neurogenic tumor of the posterior mediastinum. Pleural effusion is a rare presentation of benign schwannoma and it is mainly related to malignant tumors. Histologically, schwannoma as well as solitary fibrous tumor should be considered as a differential diagnosis of spindle cell lesions.

**Case presentation:**

Here, we report a case of an asymptomatic 61-year-old female misdiagnosed as solitary fibrous tumor of posterior mediastinum which was revealed to have blood stained pleural effusion during the video-assisted thoracic surgery. Eventually pathological study and immunohistochemistry profile of the tumor was reported as benign schwannoma.

**Conclusion:**

This report indicates that benign schwannoma can be accompanied by bloody pleural effusion and it also emphasizes the role of immunohistochemistry in the diagnosis of biopsy specimen of spindle cell lesions.

Schwannoma is a rare, typically benign, slow-growing and asymptomatic peripheral nerve sheath tumor derived from schwan cells. It occurs more often in the third to fourth decades and affects both genders equally (1). It is considered to be the most common neurogenic tumor of the posterior mediastinum (2). Malignant transformation in schwannoma is extremely rare (3). Although benign schwannomas are usually asymptomatic, Common presentations are pain and neurogenic symptoms (1). Pleural effusion is mostly associated with malignant schwannoma which is a rare condition in benign schwannoma (4). Histologically, schwannoma and solitary fibrous tumor are both considered as differential diagnosis of spindle cell lesions (5). Although they have different patterns, diagnosis of biopsy specimen is challenging if not considering immunohistochemistry (6). Here, we report a case of incidentally diagnosed benign schwannoma with blood stained pleural effusion detected during surgery. The core needle biopsy specimen was misdiagnosed as solitary fibrous tumor.

## Case Presentation

**Figure 1:**
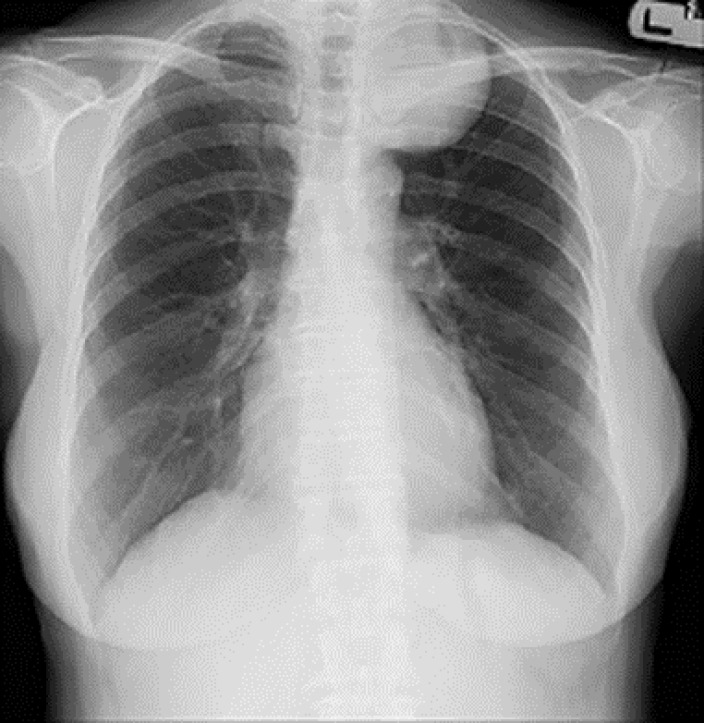
Chest radiograph showing a well-circumscribed spherical opacity occupying the upper part of the left hemithorax

A previously well 61-year-old female presented with 2 weeks of pain in her right shoulder during deep inspiration. Unexpectedly, physical examination revealed decreased breath sound in the apex of the left lung. Chest radiograph showed a well-defined round shaped opacity at the top of the left hemithorax (Fig1).

**Figure 2:**
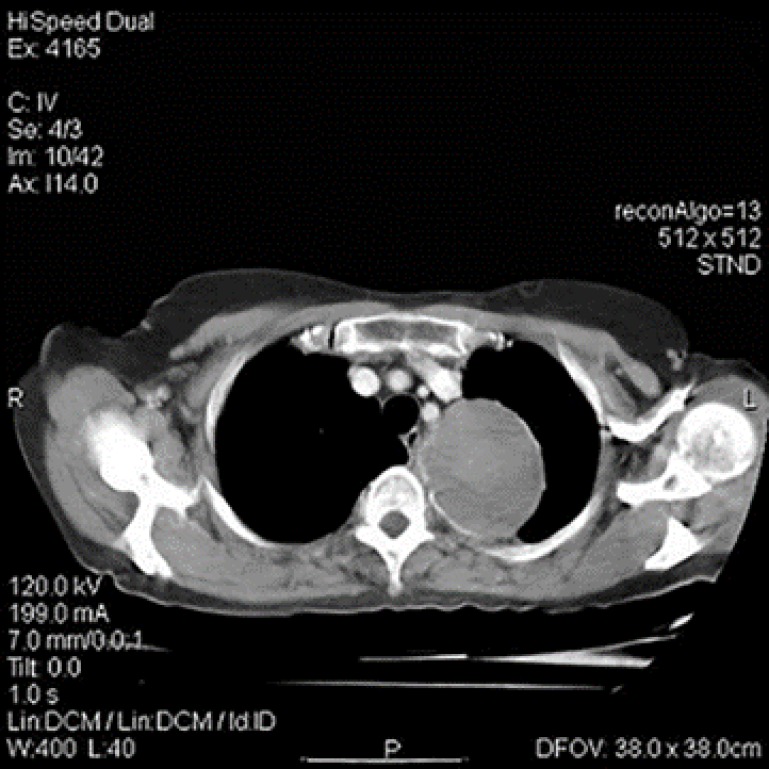
Axial computed tomography showing a large round heterogenous mass with smooth border at mediastinal boarder of the left hemithorax

Computed tomography (CT) showed a round, encapsulated, heterogeneous mass in the left upper hemithorax at the mediastinal border (Fig 2). 

**Figure 3:**
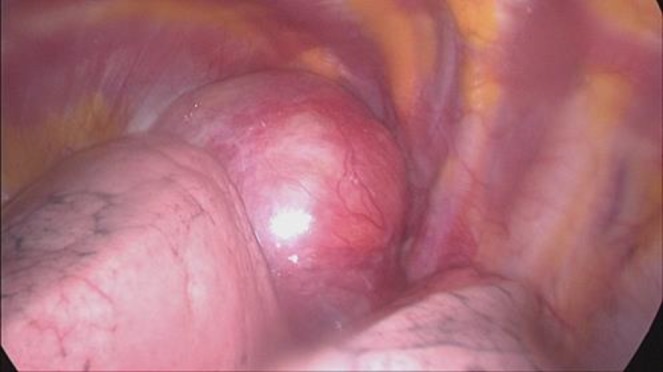
Video-assisted thoracoscopic view showing a large round-shaped neurogenic tumor adherent to the lung at the posterior

The patient underwent CT-guided core needle biopsy, which was reported as a benign appearing spindle cell lesion resembling solitary fibrous tumor of pleura. She underwent left sided video-assisted thoracic surgery with a probable diagnosis of solitary fibrous tumor (Fig3). During the procedure, 20 cc of blood stained pleural fluid was seen and aspirated. The tumor arose from the first intercostal nerve on the posterior aspect of the chest wall. 

**Figure 4:**
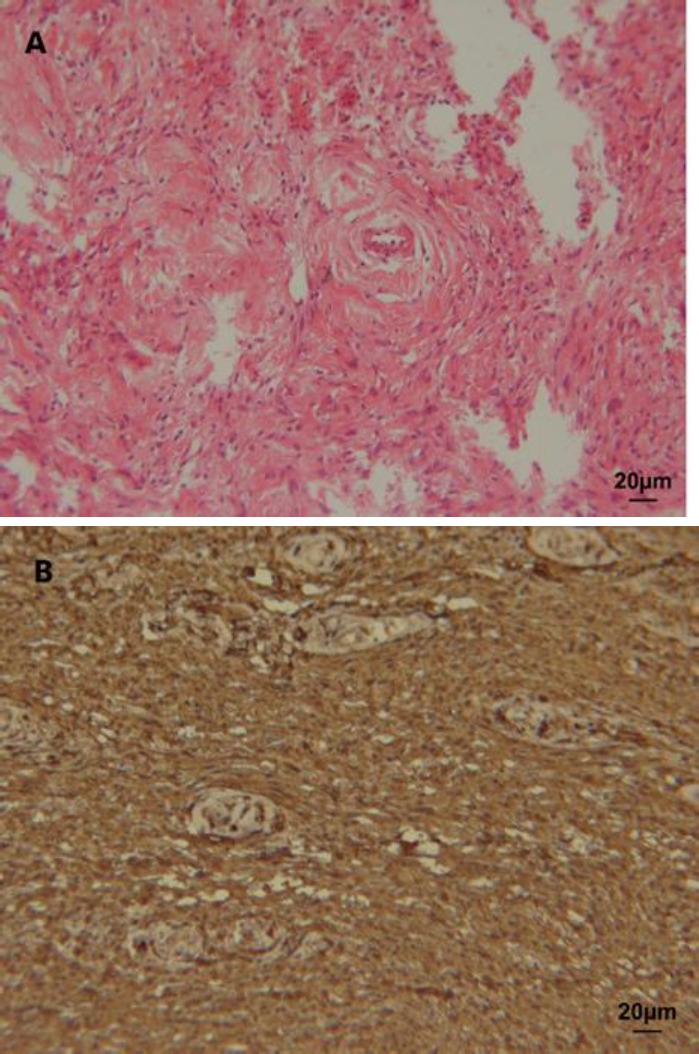
A Microscopic findings of hematoxylin-eosin stained section of the tumor showing clusters of spindle cells with uniform nuclei consistent with Antoni type A and B areas, viewed using a compound light microscope (400x) b Immunohistochemistry showing S100 positivity of tumor cells, viewed using a compound light microscope (400x)

The tumor was completely resected and carefully detached from the intercostal nerve. Since the apical segment of the left upper lobe had a pathological appearance, wedge resection of the left upper lobe was done. Macroscopically, the tumor was a heterogeneous, encapsulated, partially lobulated mass with a total size of 59×72×63 mm. Histological features of the tumor was consistent with benign schwannoma and immunohistochemical staining for S100 protein was positive (Fig 4a, b). 

The pathology of the upper segment of the left upper lobe was reported as beginning of hemorrhagic infarct and pleural fluid cytology revealed bloody smears with large sheets and clusters of atypical cells. The patient was fully recovered and discharged 5 days postoperatively. She was followed up for 9 months and further investigations revealed no source of malignancy.

## Discussion

Intrathoracic neurogenic tumors are uncommon but they compromise 75% of primary posterior mediastinal neoplasms and approximately 90% of them occur in the posterior mediastinum (2). Schwannomas, also known as neurilemomas, and neurofibromas are known as the most common mediastinal neurogenic tumors (7). Schwannomas are mostly asymptomatic and therefore usually are diagnosed incidentally. Few patients may experience paresthesia or pain due to intraspinal tumor extension or compression of adjacent structures. In this case, the pain she experienced did not seem to be related to the tumor since it was on the opposite side of the tumor and self-limited before any interventions. Signs and symptoms such as pain, dyspnea, pleural and precardial effusion have been reported to be mostly associated with malignant schwannomas (8-11). In a recent study, out of 49 mediastinal benign schwannomas only 1 patient presented with pleural effusion. Although they were mostly asymptomatic and diagnosed incidentally, the most common presentation was pain (11). 

In the same study, pain, cough, dyspnea, shortness of breath and pleural effusion was reported as common symptoms of malignant peripheral nerve sheath tumors (11). Apparently in all reported cases of benign schwannoma with pleural effusion the effusion was blood stained, therefore spontaneous tumor hemorrhage has been suggested as one of the possible etiologies of effusion (4). In this case, pleural effusion was not massive, hence it had no symptoms and had not even been detected in imaging.

Histologically, schwannomas are circumscribed spindle cell neoplasms composed of hypercellular and hypocellular areas called Antoni A and Antoni B patterns respectively, and are positive for S100 protein (12). The pathology of core needle biopsy in this case was reported as spindle cell lesion resembling solitary fibrous tumor. Solitary fibrous tumors are spindle cell neoplasms arranged in a patternless pattern (13). Since some distinctive features of solitary fibrous tumor are not easily detectable in core needle biopsy specimens, diagnosis is more challenging (6). Solitary fibrous tumors are positive for CD34 but do not express S100 protein which emphasizes the role of immunohistochemistry in differential diagnosis (13). But since the choice of treatment is the same in benign soft tissue neoplasms, it may be less critical to differentiate them. This report confirms that benign schwannomas can be accompanied by blood stained pleural effusion. It also emphasizes the role of immunohistochemistry in differential diagnosis of spindle cell lesions.
